# Synovial Cysts of the Temporomandibular Joint: An Immunohistochemical Characterization and Literature Review

**DOI:** 10.1155/2013/508619

**Published:** 2013-03-20

**Authors:** B. Vera-Sirera, J. A. Tomás-Amerigo, C. Baquero, F. J. Vera-Sempere

**Affiliations:** ^1^Department of Stomatology, School of Medicine and Dentistry, University of Valencia, 46010 Valencia, Spain; ^2^Service of Maxillofacial Surgery, La Fe University Hospital, 46009 Valencia, Spain; ^3^Department of Pathology, La Fe University Hospital and School of Medicine and Dentistry, University of Valencia, 46009 Valencia, Spain

## Abstract

Synovial cysts of the temporomandibular joint (TMJ) are very rare, and to date, only 12 cases of a synovial cyst in the TMJ region have been reported in the literature. In this paper, we present the clinicopathological and immunohistochemical characteristics of one such lesion affecting a 48-year-old woman, presented with a mass in the left preauricular region. We describe the usefulness of immunohistochemical analysis for recognizing the synovial lining, which allowed for clear differentiation between ganglion and synovial cysts. Immunohistochemical analyses can be used to diagnose synovial cysts with certainty; however, using at least two markers is advisable to distinguish the two existing synovial cell subtypes. Our findings indicate that synovial cysts of TMJ possess an internal lining dominated by type B (fibroblast-like) synoviocytes.

## 1. Introduction

Ganglion and synovial cysts are expansile, fluid-filled lesions of the joints, located mainly in periarticular areas of wrists, knees, and feet [1]. Such lesions in the temporomandibular joint (TMJ) are rare [2, 3], and a clear distinction between these two entities is often not established in publications in which the histopathological findings are not reported precisely enough to make a definitive diagnosis.

Synovial cysts are true synovial-lined cysts that may arise from displacement and/or herniation of the synovial lining, possibly because of increased intra-articular pressure [3]. Ganglion cysts are pseudocysts with a fibrous connective tissue wall and myxoid degenerative changes that lack a synovial cell linning and connection with the joint cavity [2]. Despite these theoretical differences, the two types of lesions are nearly always indistinguishable clinically [4] and by imaging [5]; thus, the exact nature of the pathology remains unknown until the presence or absence of a synovial lining can be demonstrated by histological analysis.

To date, only 12 cases of a synovial cyst in the TMJ region have been reported in the literature [5–15]. In this paper, we present the clinicopathological and immunohistochemical characteristics of one such lesion, and we describe the usefulness of immunohistochemical analysis for recognizing the synovial lining, which allowed for clear differentiation between ganglion and synovial cysts.

## 2. Case Presentation

A 48-year-old woman visited the oral and maxillofacial surgery clinic of our university hospital complaining of a mass in the left preauricular region, located anterior to the tragus, which was about 2 cm in diameter. The patient indicated that the mass had been growing slowly for approximately 6 months. She reported occasional dull pain, which was sometimes aggravated by opening of the jaw and chewing. The patient denied any recent trauma to the ear or mandible. Her past medical and surgical history included essential hypertension and a hysterectomy for multiple myoma. She was currently taking enalapril, amitriptyline, and tetrazepam. No history of allergic phenomena or adverse drug reactions was noted, and the patient denied tobacco, alcohol, or drug use.

**Figure 1:**
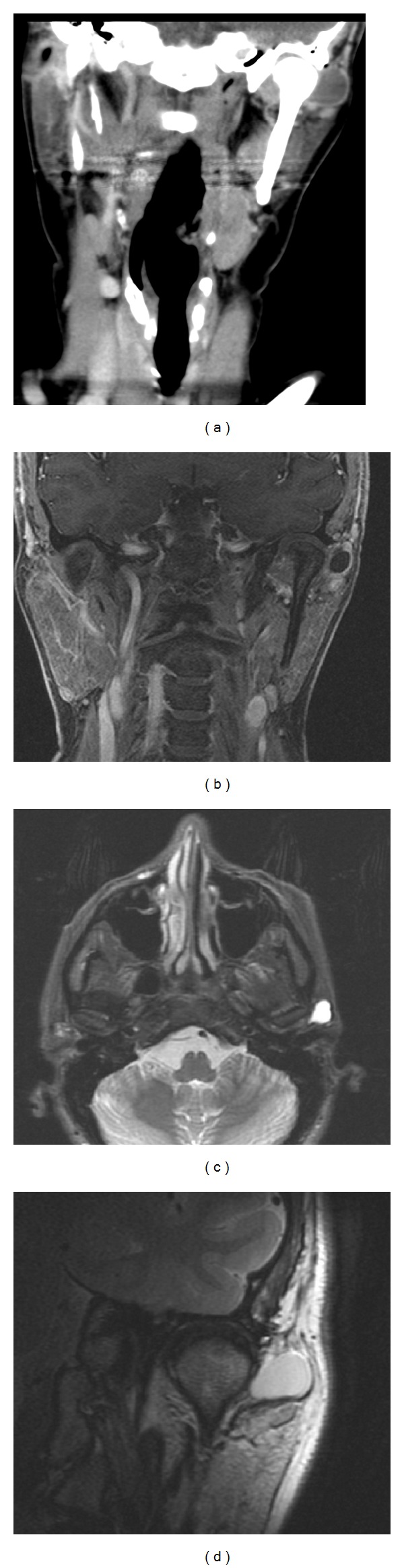
Imaging findings based on the coronal reconstruction of CT (a) and MRI sections (coronal with Gd-DTPA (b), axial STIR (c), and coronal FSE-T2 (d)) showing the features of a cystic lesion adjacent to the left TMJ.

A physical examination revealed a soft, nonerythematous, preauricular swelling (2 cm in size) in the left preauricular area lateral to the TMJ. The patient exhibited normal mouth opening, without deviation, and the mandibular range of motion was normal in all directions. No facial nerve paralysis or paresis was observed, and the rest of the head and neck examination was unremarkable. A routine panoramic radiograph showed no abnormalities. A computed tomography (CT) scan was then obtained, which revealed a hypodense mass of soft tissue of 18 × 17 × 14 cm in the left temporomandibular region (adjacent to the upper edge of the parotid gland, immediately preceding the ear and lateral to the TMJ), which did not show apparent degenerative or inflammatory changes. Likewise, magnetic resonance imaging (MRI) revealed a well-delimitated preauricular cystic lesion (located in the closed-mouth position lateral to the condyle, displacing the parotid gland inferiorly and presenting some internal septation), with the pedicle in relation to the left TMJ capsule. The position of the articular disk and mandibular condyle displacement after mouth opening was normal, and the mandibular condyle showed no morphological changes. The imaging-based diagnosis was a TMJ cyst (possible synovial cyst versus a ganglion cyst in the TMJ; Figure 1), and surgical intervention was recommended to the patient. Two weeks before surgery, fine needle aspiration revealed a proteinaceous amorphous material with occasional histiocytes and an absence of inflammatory, epithelial, or lymphoid cells.

Under general anesthesia, a preauricular approach to the left TMJ was used. The facial nerve trunk and its branches were identified, and the cystic lesion above the frontal branch of the nerve was isolated. The entire cystic lesion was removed after careful dissection of the pedicle. At 6 months after surgery, the patient showed normal mandibular joint function and no recurrence of preauricular swelling, pain, or facial nerve injury.

**Figure 2:**
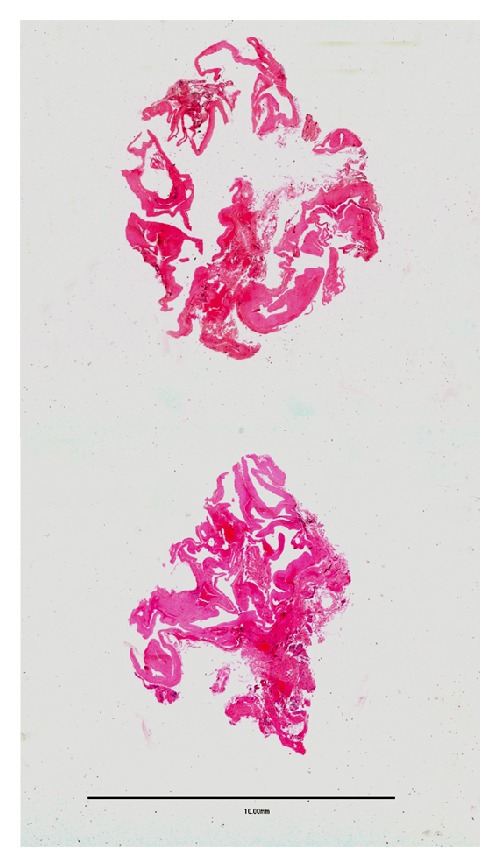
Panoramic view of the lesion in two serial histological sections showing a multilocular cyst (bar: 10 mm) (hematoxylin and eosin: 1x).

**Figure 3:**
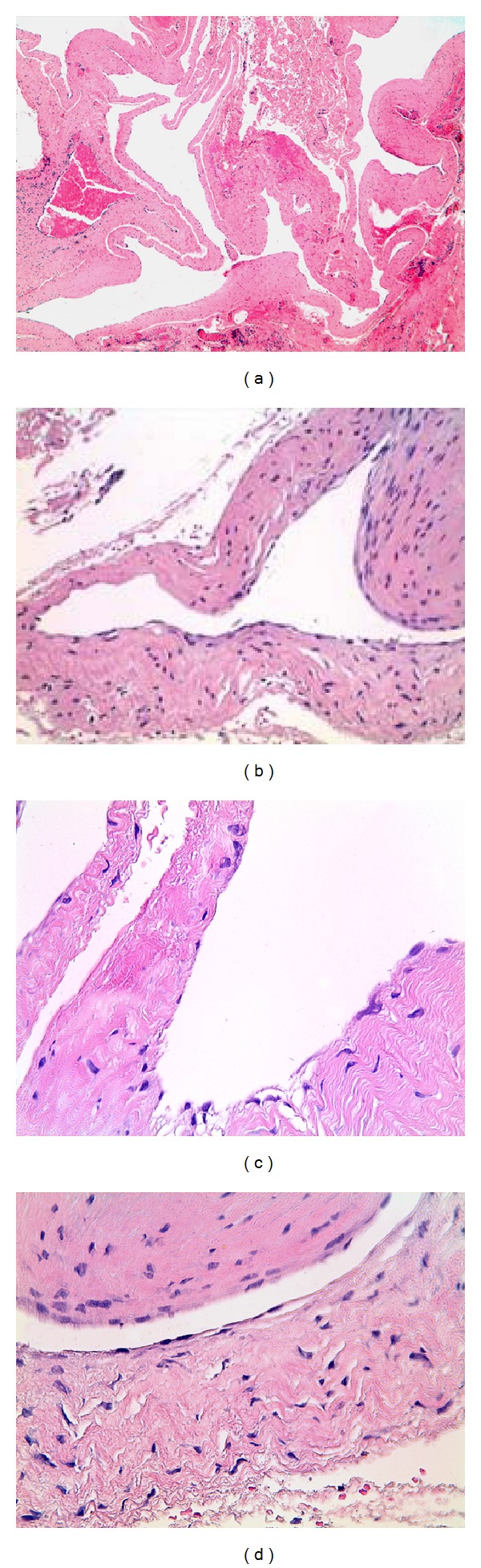
Histologically excised lesion showing a multilocular cyst (a) with fibrous walls. Each of the cystic cavities was lined with a layer of attenuated elongated cells (b), often arranged discontinuously (c) and frequently imperceptible or difficult to recognize (d) (hematoxylin and eosin: 25x, 100x, 250x, and 400x).

The surgical specimen obtained was fixed in 10% formalin and sent for pathological study. Histological examination of the excised mass showed a multilocular cystic lesion (Figure 2) discontinuously lined by attenuated cells with a fusiform or round nucleus, sometimes with a synovial appearance. This lining was often very discontinuous, and in many areas of the cystic structure, the existence of an inner lining was imperceptible or barely recognizable. The cyst wall exhibited a stromal connective core with spindle cells that were fibroblastic in appearance, sometimes similar to synovial fusiform cells but occasionally also associated with myxoid stromal changes (Figure 3).

To identify the structural features of the synovial membrane and establish with certainty the presence of synoviocytes in the lining of the cyst, we performed an immunohistochemical analysis using a panel of eight antibodies (Table 1). All immunostaining was performed using the EnVision Flex Plus visualization system (Dako, Glostrup, Denmark) in a Dako Autostainer Link Plus, according to the manufacturer's recommendations. Appropriate positive and negative controls were used throughout.

**Figure 4:**
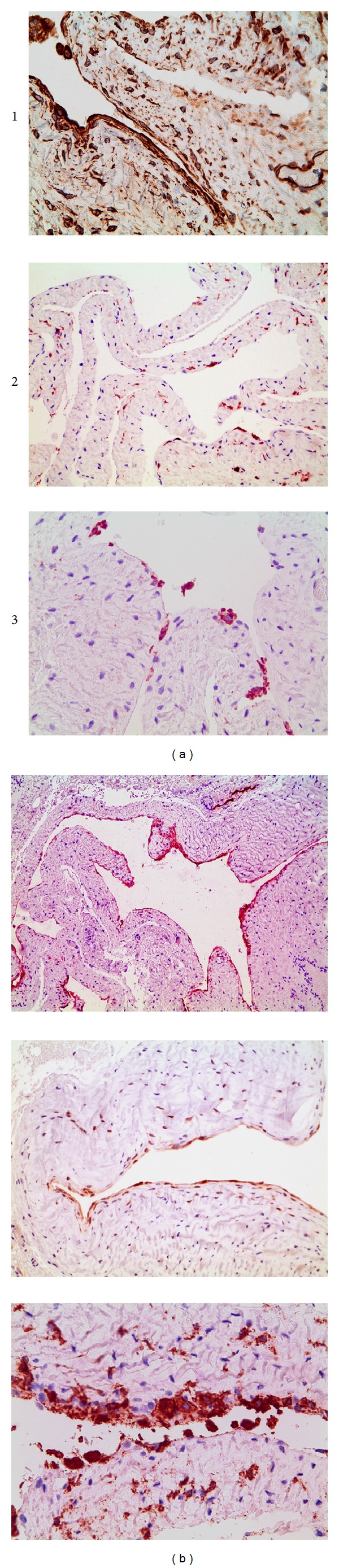
Immunohistochemical staining against vimentin (a1), D2-40 (b1), HSP-70 (a2), metallothionein (b2), CD68 (a3), and HLA-DR (b3) showing the immunoreactivity profile of the synovial cyst, with a lining of predominantly spindle-shaped cells (vimentin, D2-40, HSP-80, and metallothionein positive; type B synoviocytes) and a small population of CD68^+^ cells (type A synoviocytes) highlighted by HLA-DR staining (VT: 250x; D2-40,200x; HSP-70: 200x; metallothionein: 250x; CD68: 250x; HLA-DR: 400x).

On immunohistochemical examination, we observed vimentin staining of the intimal lining of the cyst, but this marker was also positive in other mesenchymal structures of the cystic wall, indicating reduced specificity. Of the remaining immunostains employed, D2-40 (podoplanin) was found to be the most useful for highlighting the synovial lining of the cyst; it clearly marked the entire inner contour of the cyst, excluding the possible existence of a ganglion cyst (Figure 4). The synovial cells positive for D2-40 were predominantly fusiform and preferentially of an intimal location; thus, they were identified as type B (fibroblast-like) synoviocytes. In contrast, strong CD68, lysozyme, and HLA-DR signals were observed in a variable number of synovial cells located at the intimal and subintimal levels, with a rounded contour and a macrophagic appearance (i.e., type A synoviocytes).

Taken together, our immunohistochemical findings indicate that the synovial cyst had a clear predominance of elongated type B (fibroblast-like) synoviocytes, which were clearly immunoreactive to D2-40, compared with type A (macrophage-like) synoviocytes, which were few in number and displayed a rounded appearance and CD68, lysozyme, and HLA-DR positivity. Likewise, the type B synoviocytes, which predominated at the intimal and subintimal synovial levels, showed clear reactivity against heat shock protein (HSP) 70 and metallothionein together with weak cytoplasmic *β*-catenin expression.

## 3. Discussion 

The TMJ is a synovial joint that plays a crucial role in complex jaw movements. Mass lesions that can occur in the TMJ include some rare pseudotumor cystic lesions such as synovial and ganglion cysts, which are most commonly seen in other periarticular locations of the body [3, 5].

Synovial and ganglion cysts of the TMJ are rare; therefore, we could only find 12 cases of synovial cysts in the TMJ in the literature (Table 2) and only 26 published cases of ganglion cysts [2]. Considered together, the published cases of synovial and ganglion cysts indicate a predominance among female patients [3], but this prevalence was not observed when considering only synovial cysts, although drawing conclusions is difficult because synovial and ganglion cysts are often misidentified [3, 13]. Both types of cystic pathologies of the TMJ are difficult to diagnose preoperatively [4, 5] and are sometimes misdiagnosed as other cystic lesions or tumors of the parotid gland. In addition, a clear pathological distinction between synovial and ganglion cysts is not often made in reported cases; thus, these terms are used interchangeably despite their different origin and histologic characteristics.

A synovial cyst is a true cyst lined by synovial cells (synoviocytes) that may or may not communicate with the joint cavity [2], unlike ganglion cysts, which are pseudocysts lined with fibrous connective tissue [10] and not connected with the joint cavity. Therefore, ganglion and synovial cysts are histologically separate lesions [3], and the key to distinguishing between them is to identify the presence or absence of synoviocytes in the lining of the lesion. This identification, however, is not an easy task for a pathologist, given the morphological plasticity of synovial cells and our limited knowledge regarding the immunohistochemistry of the synovial membrane, particularly in normal subjects [16]. 

 Prior to our study, reported cases of synovial cysts of the TMJ usually not used immunohistochemical tools, raising sometimes doubts about the accuracy of histological diagnosis. Recently, Spincia et al. [15] illustrate the use of anticalretinin to diagnose the synovial linning, but erroneously these authors attributed an epithelial character to this linning. Likewise, Nahlieli et al. [17] showed an alleged case of ganglion cyst and introduced more confusion on this topic, claiming that a negativity against cytokeratin and S-100 protein, as well as a vimentin positivity, excluded the diagnosis of synovial cyst. Against these observations, our study wanted to emphasize the need to use a well-designed panel of antibodies to recognize the cyst linning and to diagnose accurately.

The synovium is lined by a layer of cells, variously called lining cells, intimal cells, or synoviocytes, which have historically been subdivided into type A and B cells [18]. Ultrastructural and immunohistochemical studies have shown that type A synoviocytes are macrophages or macrophage-like, while type B synoviocytes are fibroblast-like [18, 19], with only some studies analyzing in detail the normal morphology and function of the synovial membrane in the TMJ [20].

In the last decade, different immunohistochemical markers have been proposed for the identification of synoviocytes in the normal state and in various pathologies [16, 18, 20–23]; several of these were applied in our study to identify the structural features of the synovial membrane and to enable the differential diagnosis of a synovial cyst from a ganglion cyst.

Of the eight markers used in our study, D2-40 (podoplanin) was revealed to be the most useful and powerful marker for highlighting the synovial lining of the cyst and excluding the possible existence of a ganglion cyst. D2-40 is a mouse monoclonal antibody specific for human podoplanin that is often used to highlight the lymphatic endothelium. However, D2-40 reactivity has also been described in a variety of normal tissues, including the mesothelium [24] and synovial lining [22, 25], and increased expression has been reported in rheumatoid arthritis [22], essentially marking type B (fibroblast-like) synoviocytes as actively proliferating in this disease. 

Using other immunohistochemical markers (HSP-70, metallothionein, and *β*-catenin), we showed a clear predominance of type B (fibroblast-like) synoviocytes compared with type A (macrophage-like) synoviocytes, which were CD68-, lysozyme-, and HLA-DR-positive, in a synovial cyst. From a practical perspective, immunohistochemical analysis established with certainty the diagnosis of a synovial cyst; thus, using at least two different markers is advisable to distinguish the two synovial cell subtypes.

Finally, increased reactivity against HSP-70 and metallothionein was found in the synovial cyst. The HSP family protects cells under stressful conditions and is a useful marker for type B synovial cells [20, 23]. Some components of the HSP family also act as estrogen receptor-associated proteins, suggesting that the fibroblast-like type B cells in the TMJ are targeted by estrogen [20]. This could explain the higher frequency of temporomandibular disorders in females than in males, which is also observed when published cases of synovial and ganglion cysts are considered [3]. Meanwhile, metallothionein is a protein involved in metal detoxification that protects against oxidative stress. Metallothionein synthesis in joints can be induced by a variety of inflammatory mediators, with overexpression in synovial with chronic inflammatory and degenerative joint disease [26]. Its expression in the intimal fibroblastic-like cells of a synovial cyst suggests the existence of an associated inflammatory component, as indicated previously [9].

## Figures and Tables

**Table 1:** Antibodies used to identify structural features of synovial membrane.

Ab specificity	Clone	Dilution	Source
*β*-Catenin	*β*-Catenin-1	RtU	Dakopatts, Glostrup, DK
CD68	KP1	RtU	Dakopatts, Glostrup, DK
D2-40 (podoplanin)	D2-40	RtU	Dakopatts, Glostrup, DK
HLA-DR *α*-chain	TAL 1B5	1/30	Dakopatts, Glostrup, DK
HSP-70	HSP70 (W27)	1/500	Santa Cruz BT, Santa Cruz, CA, USA
Lysozyme	Poly Ly	1/400	Dakopatts, Glostrup, DK
Metallothionein	E9	1/100	Dakopatts, Glostrup, DK
Vimentin	V9	RtU	Dakopatts, Glostrup, DK

Ab: antibody; Poly: polyclonal; RtU: ready to use with envision Flex+.

**Table 2:** Reported cases of synovial cyst of TMJ in the literature.

Authors	Age (years)	Gender (F/M)	Location	Chief complaints	Treatment	IHC data	Recurrence
Janecka and Conley (1978) [6]	50	M	R	Swelling	Surgical	Not present	Not specified
Reychler et al. (1983) [7]	30	F	R	Swelling	Surgical	Not present	Not specified
Farole and Johnson (1991) [8]	22	M	Bilateral	Swelling, pain	Surgical	Not present	No
Bonacci et al. (1996) [9]	46	M	R	Swelling, pain	Arthroscopic	Not present	No
Chang et al. (1997) [10]	38	M	R	Swelling, pain episodes	Surgical	Not present	No
Chen et al. (1998) [11]	58	M	L	Swelling	Surgical	VT^+^, *α*-1-CHT^+^	Not specified
Goudot et al. (1999) [12]	65	M	L	Reduction mouthopening	Surgical	Not present	Not specified
Lomeo et al. (2000) [13]	47	F	L	Swelling, pain	Surgical	Not present	No
Moatemri et al. (2007) [14]	30	M	L	Swelling, pain	Surgical	Not present	No
Spinzia et al. (2011) [15]	45	F	R	Swelling	Surgical	Calretinin^+^	No
Okochi et al. (2012) [5]	31	M	Not specified	Swelling, pain	Not refer	Not present	No specified
Okochi et al. (2012) [5]	20	F	Not specified	Pain	Not refer	Not present	No specified

IHC: immunohistochemical; VT: vimentin; *α*-1-CHT: alpha-1-chymotrypsin.
